# Structural insights into the activation of somatostatin receptor 2 by cyclic SST analogues

**DOI:** 10.1038/s41421-022-00405-2

**Published:** 2022-05-20

**Authors:** Qing Bo, Fan Yang, Yingge Li, Xianyu Meng, Huanhuan Zhang, Yingxin Zhou, Shenglong Ling, Demeng Sun, Pei Lv, Lei Liu, Pan Shi, Changlin Tian

**Affiliations:** 1 Department of Chemistry and the First Affiliated Hospital of USTC, School of Life Sciences, Division of Life Sciences and Medicine, Joint Center for Biological Analytical Chemistry, Anhui Engineering Laboratory of Peptide Drug, Anhui Laboratory of Advanced Photonic Science and Technology, University of Science and Technology of China, Hefei, Anhui China; 2 Tsinghua-Peking Joint Center for Life Sciences, Ministry of Education Key Laboratory of Bioorganic Phosphorus Chemistry and Chemical Biology, Department of Chemistry, Tsinghua University, Beijing, China; 3 High Magnetic Field Laboratory, Chinese Academy of Sciences, Hefei, Anhui China

**Keywords:** Cryoelectron microscopy, Hormone receptors

## Abstract

The endogenous cyclic tetradecapeptide SST14 was reported to stimulate all five somatostatin receptors (SSTR1–5) for hormone release, neurotransmission, cell growth arrest and cancer suppression. Two SST14-derived short cyclic SST analogues (lanreotide or octreotide) with improved stability and longer lifetime were developed as drugs to preferentially activate SSTR2 and treat acromegalia and neuroendocrine tumors. Here, cryo-EM structures of the human SSTR2–Gi complex bound with SST14, octreotide or lanreotide were determined at resolutions of 2.85 Å, 2.97 Å, and 2.87 Å, respectively. Structural and functional analysis revealed that interactions between β-turn residues in SST analogues and transmembrane SSTR2 residues in the ligand-binding pocket are crucial for receptor binding and functional stimulation of the two SST14-derived cyclic octapeptides. Additionally, Q102^2.63^, N276^6.55^, and F294^7.35^ could be responsible for the selectivity of lanreotide or octreotide for SSTR2 over SSTR1 or SSTR4. These results provide valuable insights into further rational development of SST analogue drugs targeting SSTR2.

## Introduction

Somatostatin (SST) is a cyclic hormone release-inhibiting peptide with two similar physiologically functional subforms, namely, SST14 and SST28 (extension of SST14 at the N-terminus), that negatively regulates cell proliferation, the release of multiple hormones and cancer suppression via activation of five cognate SST receptors (SSTR1–5)^1,2^. SSTR1–5 are class A G protein-coupled receptors (GPCRs), and they can couple with inhibitory G proteins (Gi or Go) upon binding SST analogues^3^. These SSTRs are widely distributed in the central nervous system (CNS), peripheral tissues, pancreas, gut, and associated cancer cells, playing crucial roles in hormone release, neurotransmission, cell growth arrest, and cancer suppression^4–6^. The endogenous peptides SST14 and SST28 exhibit nonspecific binding to all five SSTRs. According to the analysis of sequence homology and ligand binding specificity, SSTRs can be divided into two categories: SSTRs 2, 3, 5, and SSTRs 1, 4^2,7^. Each SSTR subtype has specific pharmacological and physiological properties. For example, adrenocorticotropic hormone (ACTH) and thyroid-stimulating hormone (TSH) secretion are downregulated by SSTR2 and SSTR5. Glucagon secretion is principally suppressed by SSTR2. Both insulin and prolactin (PRL) are inhibited by SSTR5^4,8,9^. It is necessary to develop a variety of synthetic SST analogues that selectively block the release of certain hormones through different SSTR subtypes with good receptor subtype selectivity and stability. Among the five SSTRs, SSTR2 is aberrantly expressed in many cancer cells and tumor blood vessels, and it suppresses cancer growth and promoted cell apoptosis^10–15^. Accordingly, SSTR2 is an important drug target for the treatment of multiple diseases, such as neuroendocrine tumors (NETs), thyrotropinoma, and cancer^16–18^.

**Fig. 1:**
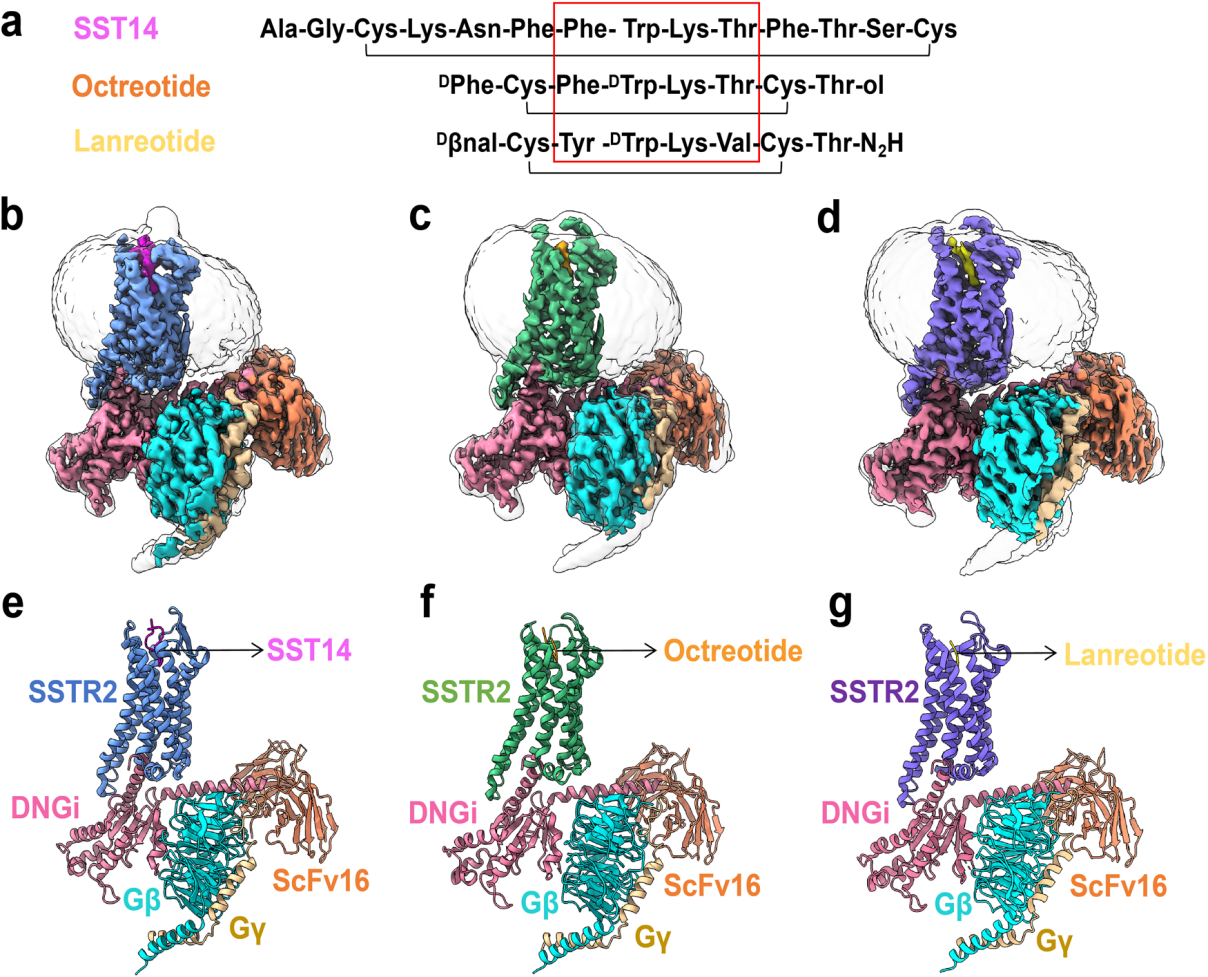
The cryo-EM structure of SSTR2–DNGi complex. **a** Peptide sequence alignment of SST14, octreotide and lanreotide. **b**–**d** Cryo-EM density maps of SST14 (**b**) or octreotide (**c**) or lanreotide (**d**) bound SSTR2–DNGi complex. **e**–**g** Ribbon diagram representation of the cryo-EM structures of SST14 (**e**) or octreotide (**f**) or lanreotide (**g**) bound SSTR2–DNGi complex, colored by subunit (TMDs in sea green, SST14 in magenta, octreotide in orange, lanreotide in yellow, Gαi in hot pink, Gβ in cyan, Gγ in gold, ScFv16 in salmon).

The endogenous peptide SST14 is a cyclic tetradecapeptide with a loop formed by a disulfide bridge between Cys^3^ and Cys^14^. The residues Phe^7^, Trp^8^, Lys^9^, and Thr^10^, which constitute a flexible β-turn, play an essential role in determining the binding affinity and biological activity of the peptide^19^. Although SST14 has the potential to modulate many pathological diseases, it has several shortcomings that have hindered its pharmacological application, including a short half-life (< 3 min) and nonselectivity (showing high binding affinity to all five SSTRs)^20^. The development of long-acting SST analogues with greater receptor subtype selectivity to regulate unique hormone release is particularly important. By using SST14 as the template, two well-known eight-residue short cyclic SST analogues (lanreotide and octreotide) were developed as commercial drugs to treat acromegalia and NETs^21,22^. The two SST analogues showed preferential binding to SSTR2, with moderate affinity to SSTR3 and SSTR5 and no affinity to SSTR1 and SSTR4^23–25^. Octreotide lacks 6 amino acids of the SST14 macrocycle but retains the three essential pharmacophore residues (Phe^3^, Lys^5^, and Thr^6^, corresponding to Phe^7^, Lys^9^, and Thr^10^ in SST14) to form β-turns. A D-amino acid (D-Trp^4^) was introduced into octreotide to replace the natural L-Trp^8^ of the core β-turn residues in SST14 to increase metabolic stability. The resulting octreotide exhibited many advantages compared with SST14, including a long half-life (2 h), reduced blood clearance, more stable conformation and improved selectivity for type 1 SSTRs (SSTR2, SSTR3, or SSTR5)^9,26^. Another SST analogue drug, lanreotide (with a half-life of 1 h), was also developed, containing the core β-turn residues (D-Trp^4^, Lys^5^) which are also present in octreotide. Moreover, two residue differences were observed in lanreotide (Tyr^3^_,_ Val^6^) versus octreotide (Phe^3^, Thr^6^) or SST14 (Phe^7^, Thr^10^) (Fig. 1a)^27^. Both lanreotide and octreotide showed specific binding to SSTR2 and played pharmacological roles in suppressing cell proliferation, cancer growth, and angiogenesis^28,29^. The two cyclic octapeptide SST analogue drugs have been widely used for clinical treatment of acromegaly caused by excessive release of growth hormone from the pituitary gland, NETs and carcinoid syndrome^30–35^. In addition, octreotide has also been approved for the treatment of TSH-secreting pituitary adenomas, liver fibrosis, refractory diarrhea and flushing^33,36,37^. Similarly, lanreotide is also clinically used to treat polycystic kidney disease, medullary thyroid cancer (MTC) and thyrotrophic adenoma with TSH secretion^38–41^. However, the structural basis for the natural biological activity of the modified SST analogues (octreotide or lanreotide) with preferential binding to SSTR2 remains unknown.

To understand the role of the two cyclic octapeptide SST analogue drugs developed from SST14 and their subtype selectivity, we herein determined the cryo-EM structures of SSTR2 in complex with inhibitory G proteins, with binding of endogenous SST14 and the two cyclic octapeptide SST analogue drugs (octreotide or lanreotide) at resolutions of 2.85 Å, 2.97 Å, and 2.87 Å, respectively. Combinatorial structural analysis and Gi-based functional data provide structural insights into the specific binding of SST analogues to SSTR2.

## Results

### Overall structures of SSTR2–Gi/SST analogue complexes

The amino acid sequences of SST14, octreotide and lanreotide are shown in Fig. 1a. All of the peptides contain homologous residues (such as Phe^7^, Trp^8^, Lys^9^, and Thr^10^ in SST14) (Fig. 1a). To investigate the molecular basis of SSTR2 activation by SST14, octreotide or lanreotide, we applied the NanoBit tethering strategy^42^ to the assembly of the SSTR2–Gi complex. An N-terminal haemagglutinin (HA) signal peptide was added to aid in the detection of the surface expression of SSTR2. A dominant-negative form of human Gαi_1_ (DNGi_1_) with two mutations (G203A, A326S)^43^ was coexpressed with Gβ_1_, Gγ_2_, and SSTR2 in insect cells. Unless otherwise specified, Gi refers to the engineered G protein, which was used for further structure determination. A single-chain antibody, ScFv16, was added to stabilize the SSTR2–Gi complex, allowing efficient assembly of the SSTR2–Gi complex. All three complexes were imaged under a Titan Krios microscope equipped with a K3 summit direct detector, and the structures of SSTR2–Gi bound with SST14, octreotide, and lanreotide were determined at global resolutions of 2.85 Å, 2.97 Å, and 2.87 Å, respectively (Fig. 1b–d; Supplementary Figs. S1–S3, Table S1).

**Fig. 2:**
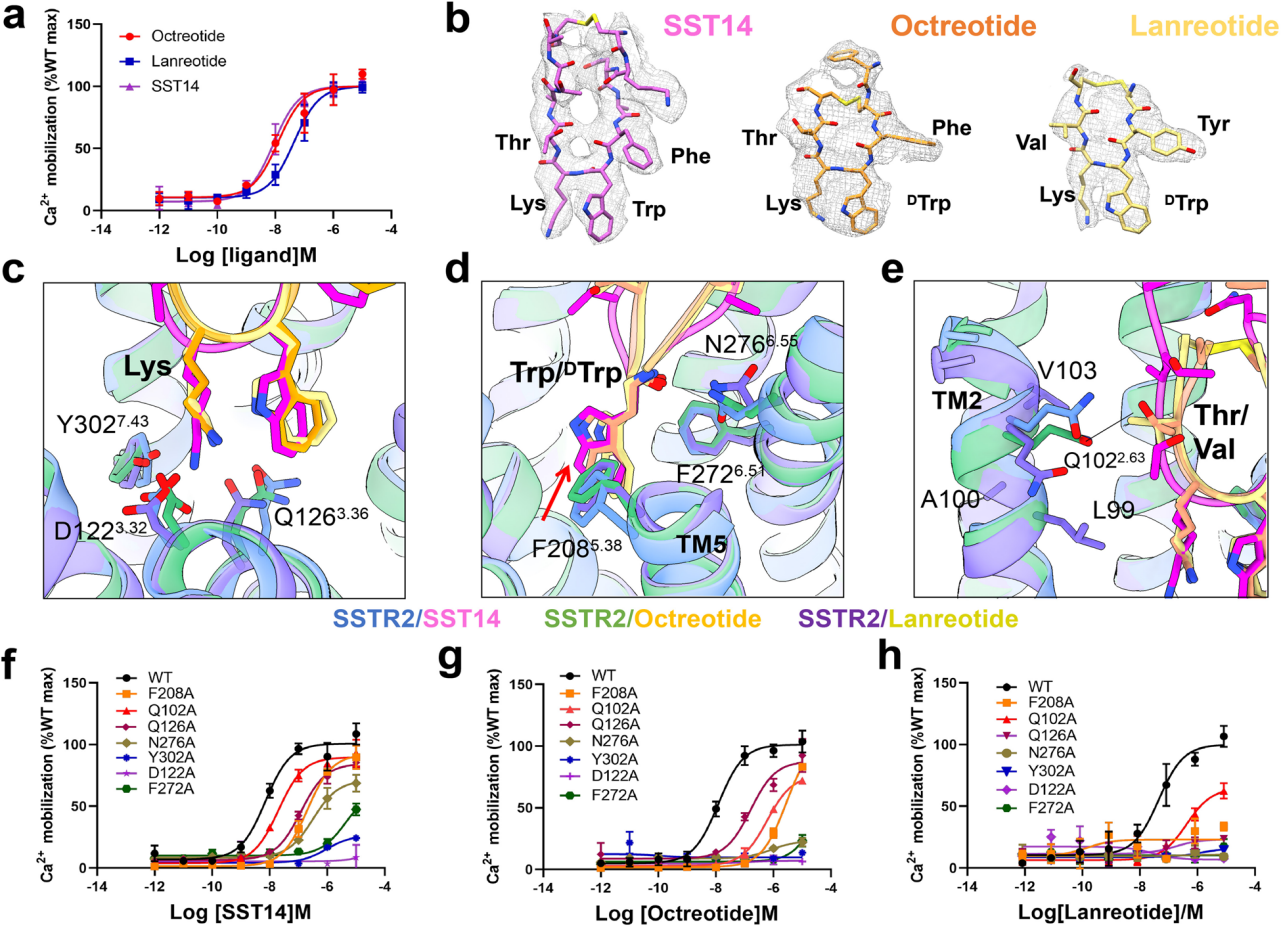
Interactions between SST β-turn residues and SSTR2 transmembrane residues. **a** Ca^2+^ response of SSTR2 with SST14, octreotide, and lanreotide. Data are presented as means ± SEM of three independent experiments conducted in triplicate. **b** Local density maps of SST14, octreotide and lanreotide. The conserved key binding amino acids are highlighted. **c**–**e** Superposition of the ligand-binding pockets of SST14- or octreotide- or lanreotide-bound SSTR2 receptor. **f**–**h** Ca^2+^ accumulation analysis of wild-type (WT) SSTR2 and mutants with SST14 (**f**) or octreotide (**g**) or lanreotide (**h**). Site mutations around the ligand-binding pocket disrupted the receptor–ligand interactions, resulting in SSTR2 malfunction in the Ca^2+^ accumulation assay. Data are presented as means ± SEM of three independent experiments conducted in triplicate.

The structures of the SSTR2–Gi complex bound with SST14, octreotide or lanreotide showed a similar overall conformation, with a root mean square deviation value of 0.55 Å for the main chain Cα atoms of the three complex structures (Supplementary Fig. S8). The high-resolution density maps showed that the majority of the side chains in the complexes were well resolved and allowed us to construct most of the regions of SSTR2 from residues T41 to V326 (Fig. 1e–g). In particular, the backbone structures of all seven transmembrane helices (TM1–TM7) of the SSTR2 receptor could be clearly defined by tracing the electron densities (Supplementary Fig. S4). The β-turn models of the three peptide ligands (SST14, octreotide or lanreotide) could also be well constructed in the ligand-binding pockets of the SSTR2–Gi complexes (Fig. 2b). All of these enabled detailed analysis of the interactions between SSTR2 and the SST analogues. Unfortunately, limited by the resolution of the N-terminal tails of the ligands, models for Ala and Gly at the N-terminus of SST14, Thr-ol at the C-terminus of octreotide, and ^D^βnal and Thr-NH_2_ at the N- and C-termini of lanreotide could not be built (Fig. 2b).

### Interactions between SST β-turn residues and SSTR2 transmembrane residues for receptor binding of the cyclic SST analogues

Despite residue deletion, modification and shorter cyclic loops in octreotide or lanreotide compared to the endogenous peptide SST14, the SSTR2 stimulation efficacy of the three peptide analogues was not greatly affected (Fig. 2a). Previous studies have shown that several β-turn residues in SST or its analogues are essential for SSTR2 binding^7,19,44^. It was reported that replacing Lys^9^ with other residues led to loss of SST14 binding to SSTR2, while changing L-Trp^8^ to D-Trp^8^ increased the metabolic stability of SST and maintained its binding affinity^45^.

Comparisons between structures of SSTR2–Gi complexes bound with the three different SST analogues (SST14, octreotide or lanreotide) reveal the detailed specific interactions between residues in the ligand-binding pockets of SSTR2 and the β-turn residues of the SST analogues, leading to structural insights into peptide ligand optimization for SSTR2 receptors with pharmacological significance. In general, the three structures all showed that the SSTR2 residues Q102^2.63^, D122^3.32^, Q126^3.36^, F208^5.38^, F272^6.51^, N276^6.55^, and Y302^7.43^ (superscripts represent Ballesteros-Weinstein numbering^46^) contributed to the interactions with β-turn residues of the SST analogues albeit with different detailed interaction specifications.

First, the essential β-turn Lys residue (Lys^9^ in SST14, Lys^5^ in octreotide or lanreotide) was observed to form a strong electrostatic interaction with D122^3.32^, cation–π interaction with Y302^7.43^, and hydrogen bond interaction with Q126^3.36^ in SSTR2 (Fig. 2c–e; Supplementary Fig. S5). The abolishment of SSTR2–Gi functions by the D122A and Y302A mutations strongly validated these interactions (Fig. 2f–h; Supplementary Table S2). The EC_50_ of SSTR2-Q126A was attenuated upon SST14 (from 10^−8^ to 10^−7^ M) or octreotide (from 10^−7.7^ to 10^−7^ M) binding, while the function of the same mutant was abolished upon lanreotide binding. These observations indicate that the interactions between β-turn Lys^5^ of lanreotide and Q126^3.36^ are essential, which is consistent with the observed closer distance between the side-chain carbonyl C(O) of Q126 and the N-H of β-loop Lys^5^ in lanreotide (3.1 Å) versus the N-H of β-loop Lys^9^ in SST14 (4.0 Å) or Lys^5^ in octreotide (3.5 Å).

Second, the β-turn Trp residue (L-Trp^8^ in SST14, D-Trp^4^ in octreotide or lanreotide) was observed to form π–π stacking or hydrophobic interactions between the indole ring of Trp and the benzyl rings of F208^5.38^ and F272^6.51^. The change from L-Trp to D-Trp enhanced the hydrophobic interaction of this residue with F208^5.38^ in the structure of octreotide- or lanreotide-bound SSTR2. The change from L-Trp^8^ in SST14 to D-Trp^4^ in octreotide or lanreotide also affected the interaction between the backbone carbonyl oxygen of the β-turn Trp and N276^6.55^ of SSTR2 (Fig. 2d). Mutagenesis experiments further supported this result, as substitution of F208^5.38^ and F276^6.55^ with alanine substantially attenuated the potency of octreotide (Fig. 2g; Supplementary Table S2), and entirely abolished the activation of lanreotide (Fig. 2h; Supplementary Table S2) but only weakly affected SST14 stimulation (Fig. 2f; Supplementary Table S2).

Third, the β-turn residue Thr^6^ in octreotide exhibited hydrogen bond interactions between the side-chain hydroxyl of Thr^6^ and the side-chain carbonyl oxygen of Q102^2.63^ (Fig. 2e), while Thr^10^ in SST14 was observed to have some distance from Q102^2.63^ (Fig. 2e). For the residue change from Thr^10^ in SST14 to Val^6^ in lanreotide, the hydrophobic side chain showed interaction with the hydrophobic residues (LAMQ^102^VAL) surrounding Q102^2.63^ in SSTR2 (Fig. 2e). Substitution of SSTR2-Q102^2.63^ with alanine was observed to substantially attenuate the potency of octreotide (EC_50_ from 10^−7.7^ to 10^−5.9^ M), but only have minor effect on SST14 function. The function data provide a clue regarding the interaction between Thr^6^ of octreotide and Q102^2.63^ of SSTR2 (Fig. 2f, g; Supplementary Table S2).

Structural and functional analysis of the interactions between the β-turn residues in SST analogues and ligand-binding pocket residues of SSTR2 showed that the β-turn Lys residue is the most essential residue and plays major roles in SSTR2 binding and receptor function stimulation. Changing L-Trp to D-Trp in the β-turn of the SST analogues not only enhanced peptide stability but also enhanced the interactions between octreotide or lanreotide and SSTR2 compared to those with SST14. Replacing the β-turn Thr to Val in lanreotide strongly changed the interactions between SST analogues and SSTR2. On the other hand, the residues in the ligand-binding pocket (Q102^2.63^, D122^3.32^, Q126^3.36^, F208^5.38^, F272^6.51^, N276^6.55^, and Y302^7.43^) of SSTR2 are essential for interactions with SST analogues.

Additionally, cross-sectional views of the ligand-binding pockets and structural superposition of SSTR2–Gi complex bound with SST14, octreotide or lanreotide showed that the extracellular loop 2 (ECL2) of SSTR2 moved downwards upon binding of octreotide or lanreotide compared with the binding of SST14 (Supplementary Fig. S6). The downwards swing of SSTR2-ECL2 enabled it to behave as a lid to make the extracellular cavity of the receptor narrower, which may further stabilize interactions between SSTR2 and octreotide or lanreotide. Similarly, a parallel study recently also reported that octreotide not only interacted closely with the transmembrane residues of the receptor but also interacted with the ECL2 of SSTR^47^.

Collectively, the modified SST14 analogues octreotide and lanreotide retained natural receptor stimulation activity similarly to that of SST14, but showed improved stability probably through the conserved interactions of the β-turn residues of octreotide and lanreotide with transmembrane residues around the ligand-binding pocket of SSTR2.

### Conformational changes in the SSTR2 receptor upon binding to lanreotide

**Fig. 3:**
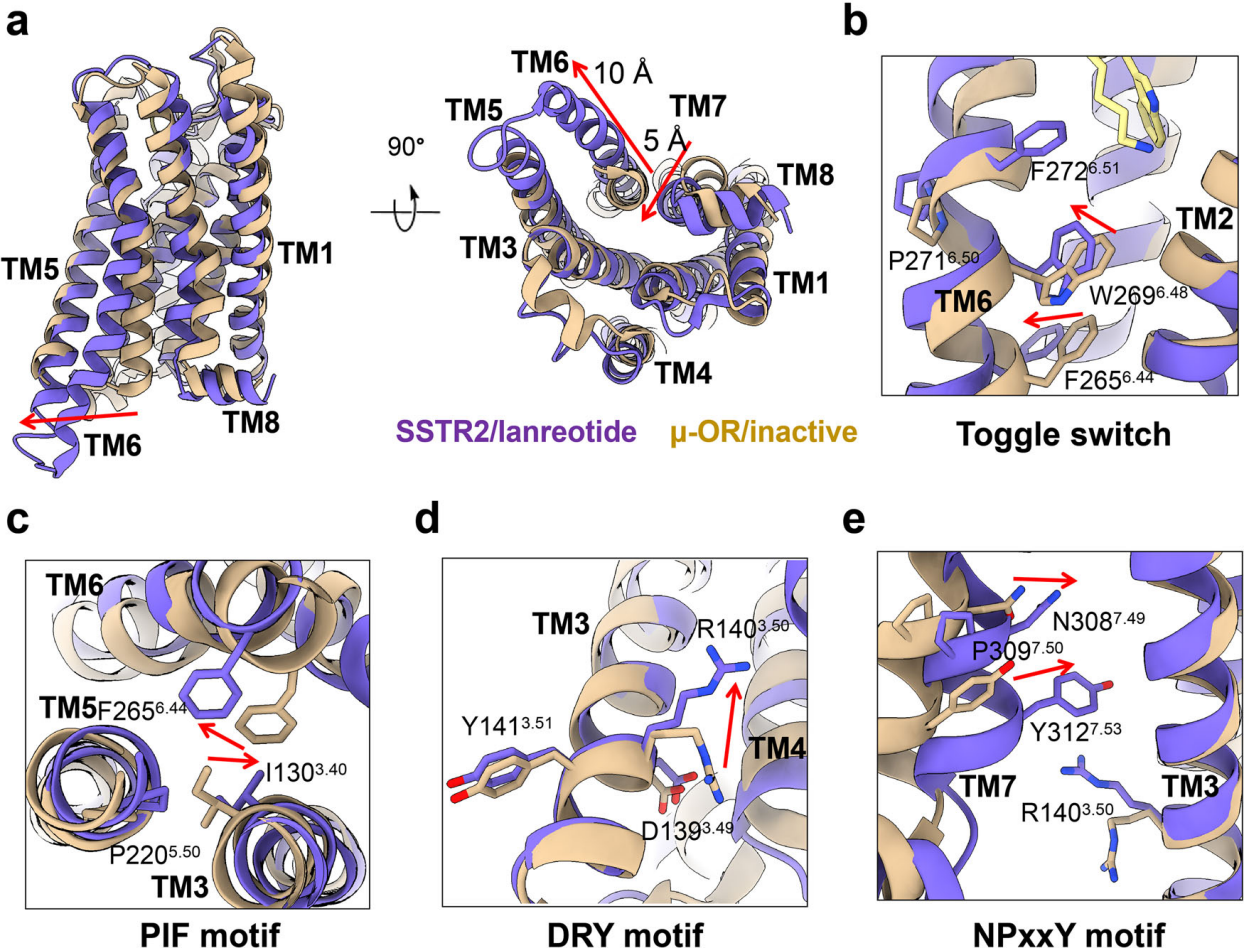
Conformational change analysis of SSTR2 receptor upon binding of lanreotide. **a** Comparision of lanreotide-bound SSTR2 (purple) and inactive state μ-OR (tan). **b**–**e** Conformational changes of toggle switch (**b**), PIF motif (**c**), DRY motif (**d**) and NPxxY motif (**e**) after SSTR2 activation upon binding of lanreotide.

Due to the absence of the structure for inactive SSTR2, a class A GPCR that shares high sequence homology with SSTR2 was applied for conformational change analysis of SSTR2 activation upon ligand binding. Among class A GPCRs, the opioid receptor was reported to have the highest sequence homology to SSTR2 (~32%) (Supplementary Fig. S7). The structure of the inactive μ-opioid receptor (μ-OR) was adopted as the reference for an inactive state. Therefore, structural superposition between SSTR2–Gi/lanreotide and inactive μ-OR (PDB: 4DKL) was implemented to analyze the structural basis of SSTR2 activation by SST analogues (Fig. 3a; Supplementary Fig. S8).

A series of conformational changes in the SSTR2 receptor were observed upon the binding of lanreotide, including structural rearrangements of highly conserved motifs that facilitate receptor activation and signal transmission from the receptor to G proteins. Compared with the inactive μ-OR, the cytoplasmic end of active SSTR2-TM6 swung downwards in two spirals and expanded outward by ~10 Å at residue V254^6.33^. In addition, the cytoplasmic end of TM7 moved inward by ~5 Å when measured at residue L315^7.56^ in the SSTR2–Gi complex/lanreotide (Fig. 3a).

Moreover, agonist binding further induced conformational rearrangements in several conserved motifs in the transmembrane domain (TMD) of SSTR2 to transmit the activation signal. Hydrophobic interactions between D-Trp^4^ of lanreotide and F272^6.51^ on TM6 could affect the “toggle switch” motif, which was located in the middle of the TMD and formed by F272^6.51^, P271^6.50^, and W269^6.48^. The interaction between D-Trp^4^ of lanreotide and F272^6.51^ rotated the angle of P271^6.50^, resulting in deflection of the side chain of W269^6.48^ (Fig. 3b) and the consequent expansion of the intracellular end of TM6. Moreover, the deflection of W269^6.48^ led to further conformational changes in F265^6.44^ and I130^3.40^ in the PIF motif and upwards movement of the R140^3.50^ side chain in the DRY motif (Fig. 3c, d). The observed upwards swinging of the R140^3.50^ side chain was considered a hallmark feature of class A GPCR activation, as previously observed in an activation analysis of the β_2_AR–Gs complex^48,49^. These conformational changes further cascaded downwards to N308^7.49^ and Y312^7.53^ in the NPxxY motif so that the cytoplasmic end of TM7 moved towards the core of the TMD (Fig. 3e). Our structures indicated that the activation of SSTR2 induced by ligand binding led to similar conformational rearrangements to other class A GPCRs^50–52^.

### Interaction interfaces between the SSTR2 and Gi protein in the presence of lanreotide

The interactions between SSTR2 and Gα_i_ are similar to those of other class A GPCR complexes. Of the three cryo-EM structures of the SSTR2–Gi/SST analogue complexes, two conventional interfaces between the transmembrane helices of SSTR2 and the α5 helix of Gi protein and between intracellular loop 2 (ICL2) of SSTR2 and the α5 and GαN helices were observed to stabilize the SSTR2 and Gi complex. Moreover, two more interaction interfaces were observed to contribute to the stability of the SSTR2–Gi complex in the presence of the SST analogue lanreotide.

**Fig. 4:**
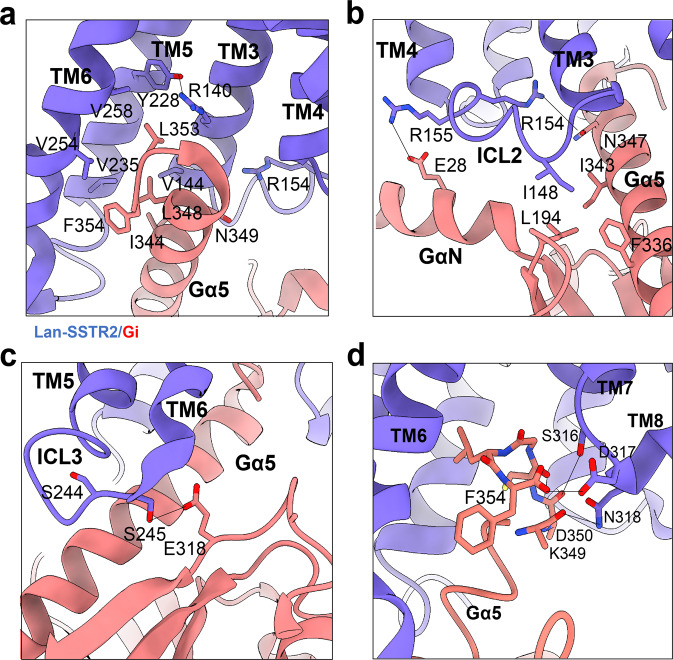
Interaction interfaces between the SSTR2 and Gi protein in the presence of lanreotide. **a** The network of interactions between lanreotide-bound SSTR2 (purple) and α5 helix of Gi (salmon). **b** The ICL2–Gi interface of lanreotide-bound SSTR2 (purple) and Gi (salmon). **c** The ICL3–Gi interface of lanreotide-bound SSTR2 (purple) and Gi (salmon). **d** The TM7–TM8 loop and α5 helix interface of lanreotide-bound SSTR2 (purple) and Gi (salmon).

The first conventional interface was observed in the α5 helix of the Gi protein, which was inserted into the central cytoplasmic cavity of the transmembrane bundle formed by TM3, TM5 and TM6 (Fig. 4a). The hydrophobic side of α5 helix directly interacted with the TM5 and TM6 helices, where L353 and F354 of α5 helix formed hydrophobic interactions with V254^6.33^ and V258^6.37^ of TM6. Additional hydrophobic interactions between I344 and L348 of α5 helix and V235^5.65^ on TM5 and V144^3.54^ on TM3 were observed to further stabilize the interface between the α5 helix and TM3 and TM5. Furthermore, the electrostatic interaction between R140^3.50^ and Y228^5.58^ could further stabilize the conformation of TM3 and TM5 of SSTR2.

The second conventional interface was observed to contain the ICL2 of SSTR2. The ICL2 helix was observed to tightly interact with the Gi protein, mainly through interactions with the α5 helix and GαN helix (Fig. 4b). The electrostatic interactions between R155^ICL2^ of SSTR2 and E28 of the GαN helix and the charge interaction between R154^ICL2^ of SSTR2 and N347 of the α5 helix were observed to stabilize the coupling of ICL2 to the Gi protein. Moreover, I148 of ICL2 was observed to form an extensive hydrophobic interaction network with I343 and F336 on α5 helix and L194 on the β2 sheet of the Gi protein.

Additionally, interactions between intracellular loop 3 (ICL3) of SSTR2 and the Gi protein were also observed (Fig. 4c); these interactions are usually not stable in other class A GPCR–G protein interfaces. Herein, S245 of ICL3 could participate in hydrogen bond formation with E318 of the Gi protein. Both interactions further stabilized the complex formed between SSTR2 and Gi protein.

Moreover, the TM7–TM8 loop could form strong interactions between SSTR2 and Gi protein. The three consecutive hydrophilic amino acids S316, D317, and N318 of the TM7–TM8 loop were observed to be in close proximity to K349, D350, and F354 of the α5 helix. The hydroxyls of S316 and D317 of the TM7–TM8 loop could form hydrogen bond interactions with the backbone carbonyl oxygens of D350 and F354 of α5 helix. The hydrogen bond interaction between NH_2_ of N318 and the backbone carbonyl oxygens of K349 could further stabilize the SSTR2–Gi complex (Fig. 4d).

### Selective activation of SSTR2 by octreotide or lanreotide

For the five somatostatin receptors (SSTR1–5), it has been reported that SST-mediated effects are dependent on receptor subtypes. Glucagon secretion and immune responses are primarily affected by SSTR2, whereas growth hormone secretion is inhibited by SSTR1, SSTR2, and SSTR5. The endogenous cyclic tetradecapeptide SST14 does not exhibit subtype selectivity for SSTRs, and it can bind and stimulate all five SSTRs. It was reported that the SST14-derived cyclic octapeptides octreotide and lanreotide do not activate SSTR1/4 but selectively activate SSTR2/3/5, predominantly SSTR2.

**Fig. 5:**
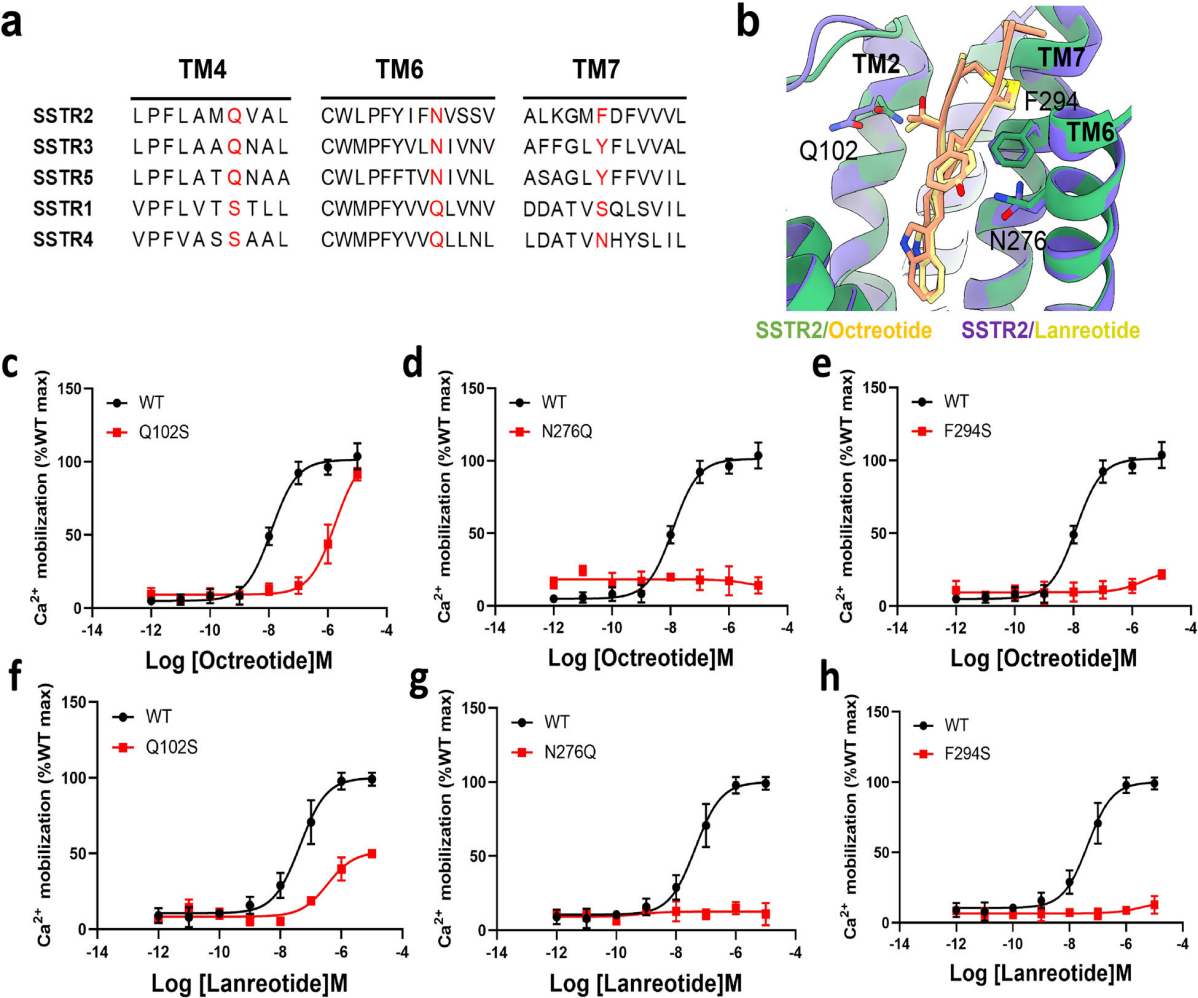
Structural and functional presentation of subtype-selective site of SSTR2. **a** Local sequence alignment of TM2, TM6, and TM7 of SSTR family. **b** Amino acid sites that may be associated with SSTR2 subtype selectivity are shown in the binding pockets of octreotide and lanreotide. **c**–**e** Ca^2+^ accumulation analysis of wild-type (WT) SSTR2 and mutants with octreotide. Site mutations to the corresponding amino acids of SSTR1 disrupted the receptor–ligand interactions, resulting in SSTR2 malfunction in the Ca^2+^ accumulation assay. Data are presented as means ± SEM of three independent experiments conducted in triplicate. **f**–**h** Ca^2+^ accumulation analysis of WT SSTR2 and mutants with lanreotide. Mutations to the corresponding amino acids of SSTR1 disrupted the receptor–ligand interactions, resulting in SSTR2 malfunction in the Ca^2+^ accumulation assay. Data are presented as means ± SEM of three independent experiments conducted in triplicate.

Structural analysis of the SSTR2–Gi complex bound with SST analogues (SST14, octreotide or lanreotide) revealed that the transmembrane residues (Q102^2.63^, D122^3.32^, Q126^3.36^, F208^5.38^, F272^6.51^, N276^6.55^ and Y302^7.43^) around the ligand-binding pockets of SSTR2 are essential for interaction with the β-turn residues of SST analogues. The sequence alignment of SSTR subtypes revealed sequence conservation and diversity in SSTR1–5 (Fig. 5a). Three residues (Q102^2.63^, N276^6.55^, F294^7.35^) in TM2, TM6 or TM7 showed distinct properties among the two SSTR types (type 1: SSTR2/3/5 or type 2: SSTR1/4).

Sequence alignment analysis of SSTR1–5 revealed that Q102^2.63^ is conserved in SSTR2/3/5 but Ser^2.63^ is conserved in SSTR1/4. In the structures of SSTR2–Gi/SST analogues, Q102^2.63^ is responsible for binding octreotide or lanreotide (Fig. 2e, g, h). Herein, the SSTR2-Q102S mutant showed a highly attenuated EC_50_ upon binding of octreotide or lanreotide versus the wild-type SSTR2 (Fig. 5c, f; Supplementary Table S2), strongly suggesting that Q102^2.63^ is important for SSTR subtype selectivity of the two cyclic octapeptide SST analogues.

In the SSTR2–Gi/SST analogue complex structures, the backbone amide N-H of N276^6.55^ formed a hydrogen bond interaction with the backbone carbonyl oxygen of D-Trp^4^ of octreotide or lanreotide (Fig. 2d, f–h). Replacing N276^6.55^ of SSTR2 with glutamine (Q), which is conserved in SSTR1/4, completely abolished the activation of SSTR2 by octreotide and lanreotide (Fig. 5b, d, g; Supplementary Table S2). These results suggest that the residue N276^6.55^ in SSTR2 might be responsible for the selective activation of SSTR2 by octreotide or lanreotide. Moreover, the mutation of F294^7.35^S in SSTR2, corresponding to residue S305^7.35^ in SSTR1, abolished the activation effect of octreotide or lanreotide on SSTR2, suggesting the crucial role of F294^7.35^ in SSTR2 activation by these two cyclic octapeptides (Fig. 5b, e, h). Furthermore, previous studies reported that N276^6.55^ and F294^7.35^ in SSTR2 could provide a more stable interaction with the disulfide bridge of octreotide and lanreotide than Q291^6.55^ and S305^7.35^ in SSTR1^53^. Some other studies have shown that substitution of the two key residues Q291^6.55^ and S305^7.35^ in SSTR1 with the corresponding residues N276^6.55^ and F294^7.35^ in SSTR2 could increase the affinity of SSTR1 to cyclic octapeptide SST analogues by up to 1000-fold^7,54^. All of these results strongly validated the essential role of N276^6.55^ and F294^7.35^ of SSTR2 in their selective interaction with octreotide or lanreotide.

## Discussion

SSTRs are widely expressed in many tissues and usually coexist in the same cell as multiple subtypes (SSTR1–5). These five receptors share common signaling pathways but are related to different physiological or pathological processes. The endogenous SST peptides SST14 and SST28 could bind and activate SSTRs with nanomolar affinity^55^. Due to the simultaneous activation of multiple targets by endogenous SST, subtype-specific SST analogues with a wide range of effects have been developed.

Successful development of cyclic octapeptide SST analogue drugs (octreotide or lanreotide) and their commercial application in hormone release arrest or cancer suppression^56,57^, together with structural analysis of the SSTR2–Gi complexes bound with SST14, octreotide or lanreotide, provided a great example for further rational optimization of SST analogues with increased selectivity for and higher metabolic stability with other SSTR members. Herein, detailed interaction analysis between transmembrane residues in ligand-binding pockets and the β-turn residues in SST analogues revealed the essential roles of the Lys (Lys^9^ in SST14, Lys^5^ in octreotide or lanreotide) and Trp (L-Trp^8^ in SST14, D-Trp^4^ in octreotide or lanreotide) residues. A parallel preprint works at *bioRxiv* also demonstrated the conserved interactions between Lys-Trp and SSTR^58^. Structural and functional analysis showed that transmembrane residues (Q102^2.63^, D122^3.32^, Q126^3.36^, F208^5.38^, F272^6.51^, N276^6.55^, and Y302^7.43^) around the ligand-binding pocket of SSTR2 are responsible for binding SST analogues for further SSTR2 activation. Compared to an inactive μ-OR, a series of conformational changes were observed for the SSTR2 receptor, from peptide–ligand interactions to transmembrane helix movements, upon its activation by SST analogues (SST14, octreotide or lanreotide). Furthermore, it was demonstrated that the interfaces between SSTR2 and Gi protein upon binding of SST14, octreotide or lanreotide were highly conserved. Stable interactions between SSTR2 and Gi protein were contributed not only by the conventional hydrophobic interaction network formed between the TM4/5/6 of SSTR2 and the α5 helix, and between ICL2 of SSTR2 and α5/αN helices, but also by electrostatic interactions or hydrophilic hydrogen bonds between ICL3, the TM7–TM8 loop of SSTR2 and the residues in Gi protein. Additionally, Q102^2.63^, N276^6.55^ and F294^7.35^ enabled selective binding of octreotide or lanreotide to SSTR2/3/5 by stabilizing the interactions between the SST analogues and type-1 SSTR (e.g., SSTR2)-specific residues.

Taken together, our structures provide a framework for better understanding the activation of SSTR2 by the endogenous cyclic tetradecapeptide SST14 and its derivative shorter cyclic octapeptide analogues (octreotide and lanreotide). Combined structural, mutagenesis and functional studies of SSTR2 and SST analogues provide insights into the subtype-selective activation of cyclic octapeptide SST analogues, which will be very valuable and helpful for further engineering of SST analogues and for drug design.

## Materials and methods

### Construct cloning

The human *SSTR2A* gene was cloned into pFastbac1 vector with the N-terminal HA signal peptide followed by a Flag tag, 10× His tag and TEV protease cleavage site. We truncated the C-terminus of SSTR2A by 10 amino acids to facilitate the better expression of the receptor. To obtain stable SSTR2–Gi_1_ complex, we used NanoBit tethering strategy, in which the C-terminus of truncated SSTR2A was attached to LgBit subunit and rat Gβ_1_ was followed by HiBit at its C-terminus. A dominant-negative human Gαi_1_ (DNGi_1_) with two mutations (G203A, A326S), Gβ_1_ and bovine Gγ_2_ were also cloned into pFastbac1 vector.

### Expression and purification of scFv16

ScFv16 with a His8 tag at C-terminus was expressed in Sf9 insect cells and purified as follows. In detail, the cells infected with scFv16 virus for 48 h were removed by centrifugation (4000 rpm, 10 min). The supernatant was purified with nickel affinity chromatography. Precipitates was resuspended in HEPES buffer (20 mM HEPES, pH 7.5, 100 mM NaCl), and then the cells were disrupted and the supernatant obtained after high-speed centrifugation (14000 rpm, 30 min, 4 °C) was also purified with nickel affinity chromatography. The Superdex 200 Increase 10/300 GL column (GE Healthcare) was used to separate the monomeric fractions of scFv16 with running buffer containing 20 mM HEPES, pH 7.5, 100 mM NaCl, 2 mM MgCl_2_. The purified scFv16 was flash-frozen by liquid nitrogen and stored at −80 °C until use.

### Expression and purification of SSTR2–Gi_1_ complex

Sf9 cells were infected with virus of SSTR2, DNGi_1_, scFv16, Gβ_1_ and Gγ_2_ at the ratio of 2:1:1:1:1:1 for 48 h at 27 °C. Cells were centrifuged (4000 rpm, 10 min), resuspended in buffer (20 mM HEPES, pH 7.4, 100 mM NaCl, 10 mM MgCl_2_, 5 mM CaCl_2_, 10% glycerol), and lysed by dounce homogenization after addition of 10 μM peptide, 0.025 U/mL Apyrase, EDTA-free protease inhibitor cocktail, 0.1 mM TCEP and 10 μg/mL scFv16. After lysis, the homogenate was incubated for 1.5 h at room temperature and then 0.5% (w/v) *n*-dodecyl-β-D-maltopyranoside (DDM) and 0.05% (w/v) cholesteryl hemisuccinate (CHS) were used for solubilization at 4 °C for 3 h. Supernatant was collected after centrifugation (45,000 rpm, 45 min, 4 °C) and incubated with Flag resin at 4 °C for 1 h. The resin was loaded onto a gravity flow column and washed with 10 column volumes of wash buffer containing 20 mM HEPES, pH 7.4, 100 mM NaCl, 10 mM MgCl_2_, 5 mM CaCl_2_, 10% glycerol, 10 μM ligand, 0.1 mM TCEP, 0.05% (w/v) DDM and 0.005% (w/v) CHS. Then the detergent was changed to 0.05% (w/v) LMNG and 0.01% (w/v) CHS and the column was washed with ten column volumes of wash buffer. The complex was eluted by 10 column volumes of elution buffer (20 mM HEPES, pH 7.4, 100 mM NaCl, 10 mM MgCl_2_, 5 mM CaCl_2_, 10% glycerol, 10 μM ligand, 0.1 mM TCEP, 0.03% (w/v) LMNG/CHS, 0.01% (w/v) GDN/CHS). The flow-through was loaded onto the Superdex 200 10/300 GL column or the Superose 6 Increase 10/300 GL column (GE Healthcare) with running buffer containing 20 mM HEPES, pH 7.4, 100 mM NaCl, 2 mM MgCl_2_, 0.00075% LMNG/CHS, 0.00025% GDN/CHS, 0.1 mM TCEP, 10 μM ligand. The peak of complex was concentrated to 0.8 mg/mL for cryo-EM sample preparation.

### Cryo-EM grid preparation and data collection

For the preparation of cryo-EM grids, 3 μL of the purified SSTR2–Gi complexes at 0.8 mg/mL were applied onto a freshly plasma-cleaned holey carbon grid (GryoMatrix-M024, R1.2/1.3, 300 mesh, Au). Grids were blotted for 10 s and plunge-frozen in liquid ethane cooled by liquid nitrogen using a Vitrobot Mark IV (Thermo Fisher Scientific) at 4 °C and with 100% humidity.

Cryo-EM imaging was performed on a Titan Krios electron microscope at 300 kV accelerating voltage using a Gatan K3 Summit direct electron detector with a Gatan energy filter in the Center of Cryo-Electron Microscopy, University of Science and Technology of China (Hefei, China). A total of 4189 movies for SSTR2–Gi/SST14 complex, 4837 movies of SSTR2–Gi/octreotide complex, and 7377 movies of SSTR2–Gi/lanreotide complex were collected with a nominal magnification of 81,000×, corresponding to a pixel size of 1.07 Å using the EPU software. Each movies stack was recorded for a total of 3.5 s and 30 frames per micrograph with a defocus range of −1.2 μm to −2.2 μm.

### Image processing and map construction

Dose-fractionated image stacks were subjected to beam-induced motion correction using MotionCor2 (v1.0.6). The processed images were transferred to CryoSPARC (v3.0)^59^ and contrast transfer function (CTF) estimation was performed with patch CTF estimation. Following CTF estimation, auto-picking particles were extracted by four-time downscaling resulting in the pixel size of 4.28 Å. After two rounds of 2D classification, the particles from well-defined 2D averages were extracted with a pixel size of 2.14 Å for further ab initio reconstruction and heterogeneous refinement. After several rounds of heterogeneous refinement, the selected subset of particles from heterogeneous refinement were extracted with a pixel size of 1.07 Å. The following homogeneous refinement and nonuniform refinement yielded a density map at 2.85 Å for SSTR2–Gi/SST14 complex, 2.97 Å for SSTR2–Gi/octreotide complex, and 2.87 Å for SSTR2–Gi/lanreotide complex.

### Model building and refinement

The initial model for the human SSTR2 receptor was derived from inactive state SSTR2, which was simulated by AlphaFold followed by extensive remodeling using COOT^60^. The DNGi_1_βγ heterotrimer was derived from μ-OR–Gi_1_ complex (PDB code: 6DDF). The N-terminal residues 1–40 and C-terminal residues 327–398 of SSTR receptor were not built due to the lack of corresponding densities. Structure refinement and model validation were performed using phenix.real_space_refine module in PHENIX^61^. The final model was subjected to refinement and validation in PHENIX. Figures were prepared using UCSF Chimera^62^ or UCSF Chimera X^63^.

### Intracellular Ca^2+^ mobilization signaling assay

SSTR2 (wild-type or mutant) and Gq_i9_ cloned in pcDNA3.1 vector were co-transfected into 293 T cells at a ratio of 2:1 with Lipo3000. After 24 h, the cells were plated in poly-D-lysine-coated 96-well plates. After cells were washed with calcium buffer (HBSS buffer supplemented with 0.1% BSA, 2.5 mM probenecid, pH 7.4), Fluo4 AM was added and incubated for 50 min in a 37 °C incubator. After washing with calcium buffer, 50 μL calcium buffer was added to each well. Fluorescence intensities were recorded simultaneously from all the wells by a FLIPR. Plates were illuminated at 488 nm and fluorescence emission was recorded at 540 nm. The fluorescence intensity was measured at 1-s intervals for 410 s. This procedure was followed by the addition of 50 μL agonist. Data were normalized to the baseline response of the ligand. Data were analyzed using nonlinear regression in GraphPad Prism software 9.0.

### Cell surface expression level analysis

Cell surface expression was determined by flow cytometry (CytoFLEX). HEK-293T cells were seeded in six-well plates 24 h before transfection at a density of 5 × 10^5^ cells in 2 mL growth medium and transiently transfected with either the wild-type SSTR2 or mutants for 24 h. After transfection, cells were centrifuged at 400× *g* for 4 min and washed with 500 μL PBS. The cells were blocked with 3% BSA in PBS at room temperature for 10 min and then incubated with anti-Flag antibody (BioLegend, 637310) at room temperature for 45 min. The cells were washed 3 times, centrifuged at 400× *g* for 4 min and resuspended in PBS. Cell surface expression was determined by quantifying PE fluorescence when gating on the live cell population using forward and side scatter. Expression levels as measured by mean fluorescence were normalized to the expression level of wild-type SSTR2. Each construct was analyzed in three independent experiments.

## Supplementary information

Supplementary information

## Data Availability

The cryo-EM density maps and corresponding atomic coordinates of human SSTR2–Gi complex bound with SST14, octreotide or lanreotide have been deposited in the Electron Microscopy Data Bank and the Protein Data Bank under the accession codes of EMD-33098, EMD-33099, EMD-33100 and 7XAT, 7XAU, 7XAV, respectively. All data analyzed in this study are included in this paper and Supplementary information.
