# Supplementary material for: Structural insights into the activation of somatostatin receptor 2 by cyclic SST analogues
Source: Cell Discov. 2022 May 20;8:47. doi: 10.1038/s41421-022-00405-2 (PMC9122944; doi:10.1038/s41421-022-00405-2)
Supplement: Supplementary file 1 — Supplementary information [file 41421_2022_405_MOESM1_ESM.pdf]

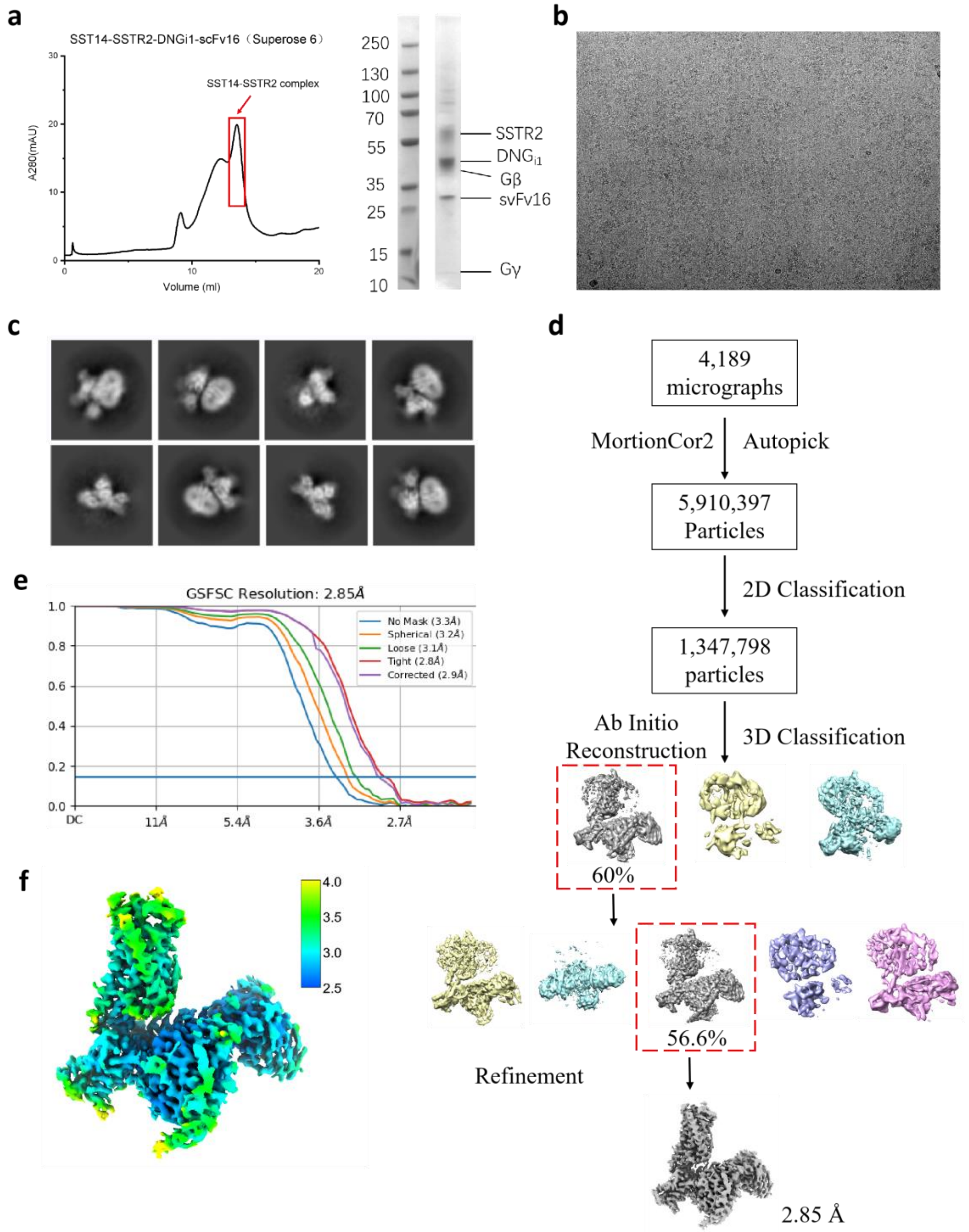

Fig. S1 Purification and cryo-EM data processing of SST14 bound SSTR2-Gi complex.

a Size-exclusion chromatography profile and SDS-PAGE analysis of SSTR2-Gi complex bound with SST14. **b** Representative micrograph after motion correction and dose weighting. **c** 2D class averages of SSTR2-Gi complex bound with SST14. **d** Flow chart of cryo-EM data processing using cryoSPARC. **e** Gold-standard FSC validation curves from cryoSPARC. **f** Density map of SSTR2-Gi/SST14 complex colored by local resolution estimation.

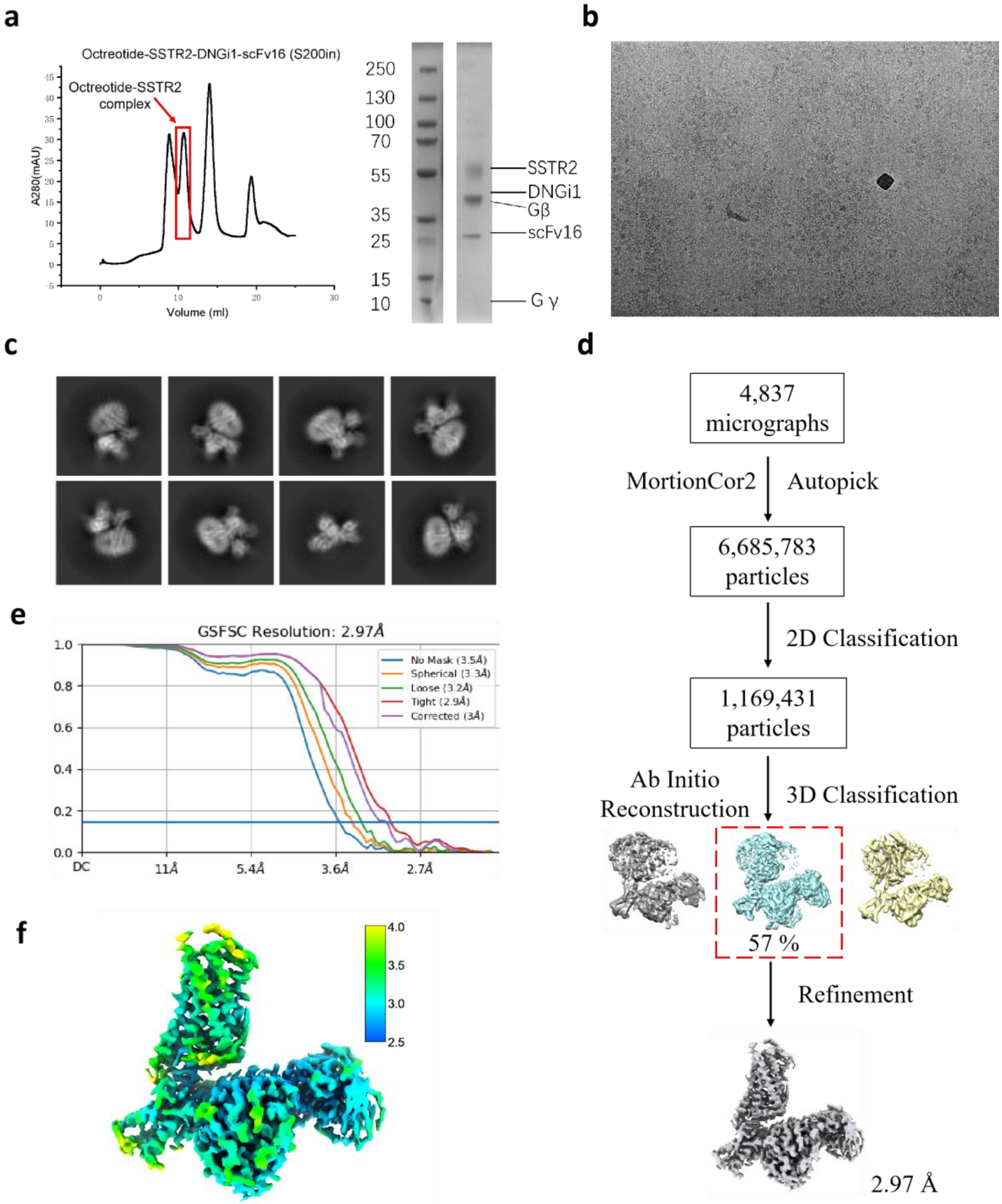

Fig. S2 Purification and cryo-EM data processing of octreotide bound SSTR2-Gi complex.

a Size-exclusion chromatography profile and SDS-PAGE analysis of SSTR2-Gi complex bound with octreotide. **b** Representative micrograph after motion correction and dose weighting. **c** 2D class averages of SSTR2-Gi complex bound with octreotide. **d** Flow chart of cryo-EM data processing using cryoSPARC. **e** Gold-standard FSC validation curves from cryoSPARC. **f** Density map of SSTR2-Gi/octreotide complex colored by local resolution estimation.

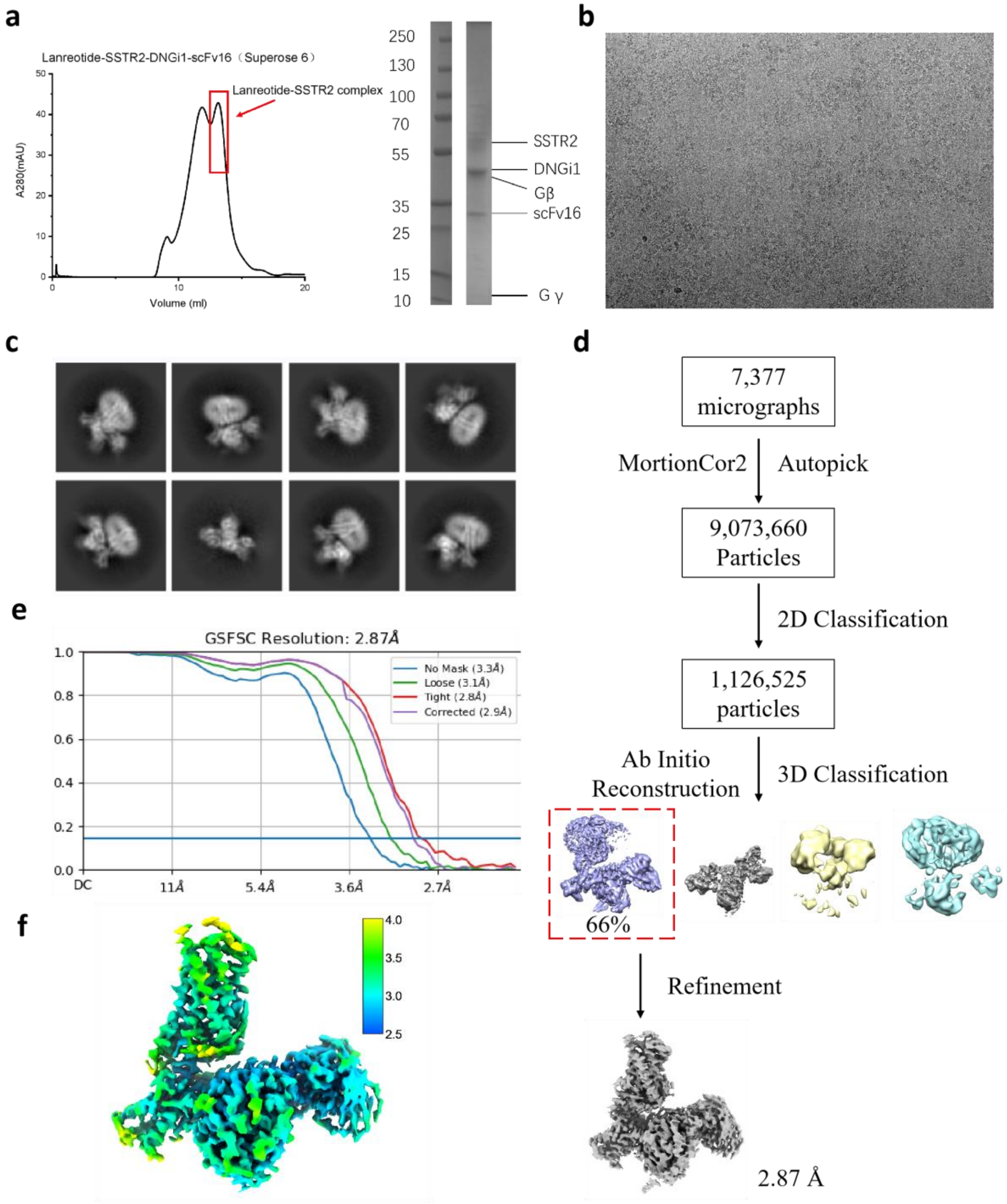

Fig. S3 Purification and cryo-EM data processing of lanreotide bound SSTR2-Gi complex.

a Size-exclusion chromatography profile and SDS-PAGE analysis of SSTR2-Gi complex bound with lanreotide. **b** Representative micrograph after motion correction and dose weighting. **c** 2D class averages of SSTR2-Gi complex bound with lanreotide. **d** Flow chart of cryo-EM data processing using cryoSPARC. **e** Gold-standard FSC validation curves from cryoSPARC. **f** Density map of SSTR2-Gi/lanreotide complex colored by local resolution estimation.

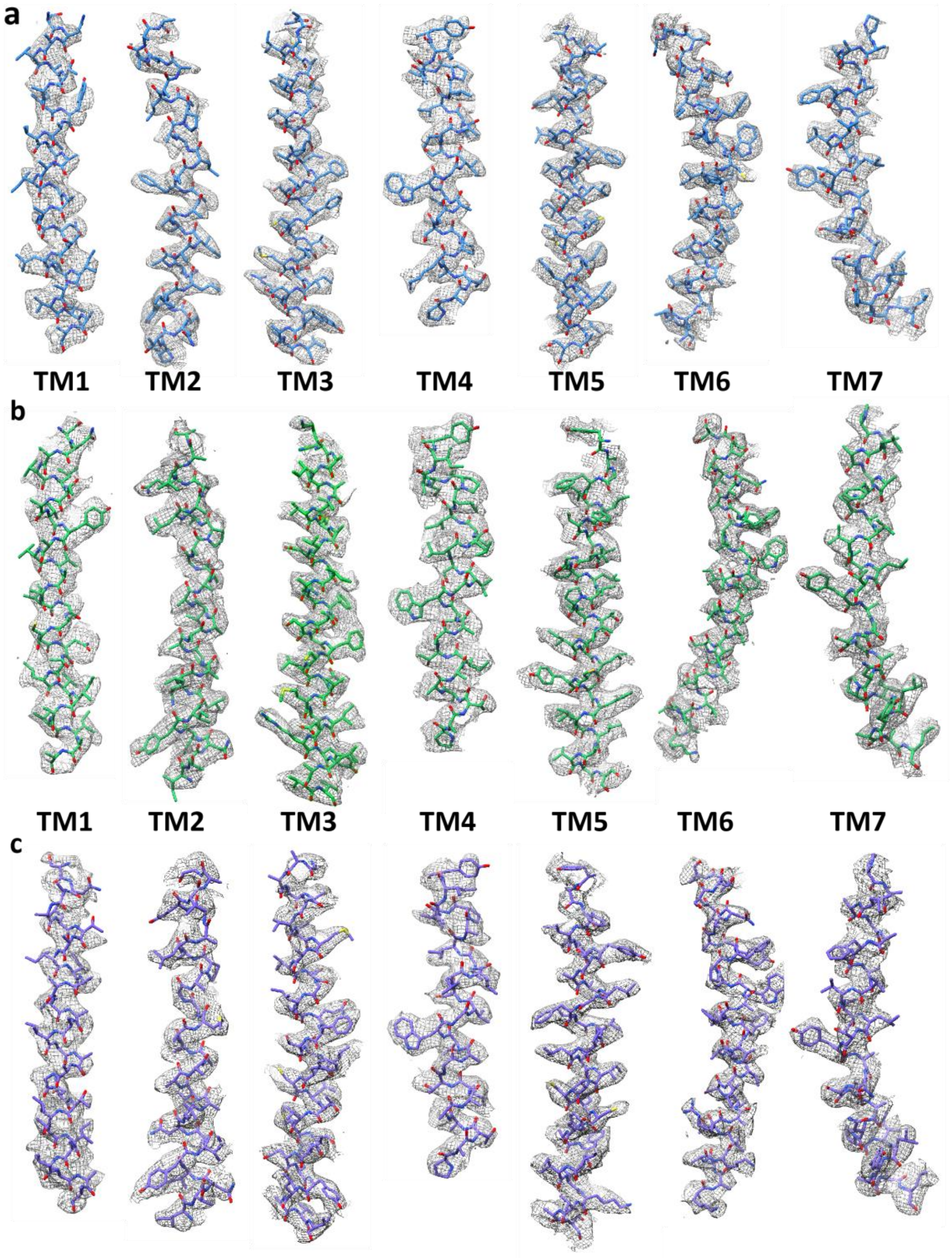

Fig. S4 Cryo-EM densities of representative segments of SSTR2-Gi complex.

a-c Cryo-EM density maps and models of transmembrane domain of SSTR2-Gi/SST14 complex**(a)**, SSTR2-Gi/octreotide**(b)** and SSTR2-Gi/lanreotide complex**(c)**. The cryo-EM maps are shown in mesh and molecular models as stick representation.

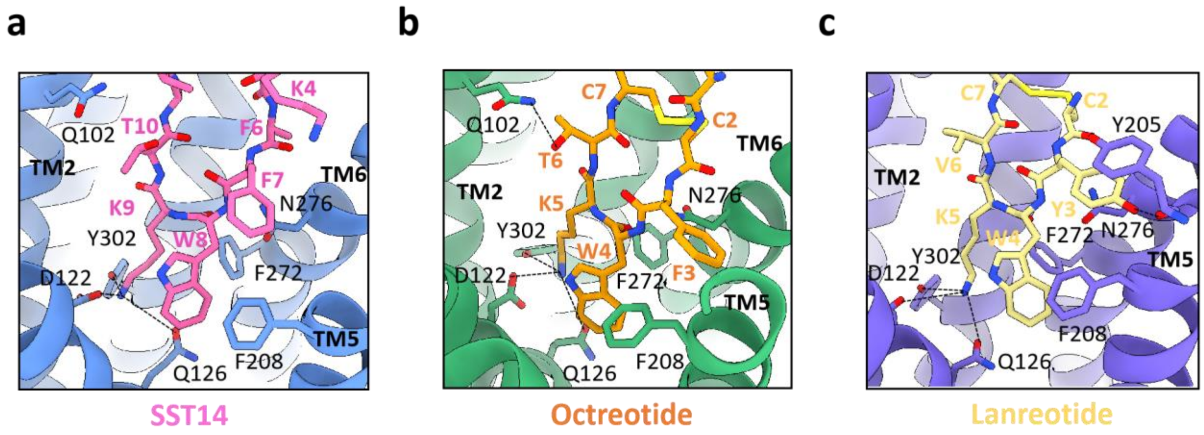

Fig. S5 The ligand binding pockets of SSTR2 bound with SST14, Octreotide or Lanreotide.

Side views of the orthosteric binding pockets in the SST14-bound SSTR2**(a)**, ortreotide-bound SSTR2**(b)** and lanreotide-bound SSTR2**(c)** structures. Residues within 4 Å of both ligands are shown as sticks and the hydrogen bond interactions are represented by dotted lines. In both panels, red and blue sticks represent oxygen and nitrogen, respectively.

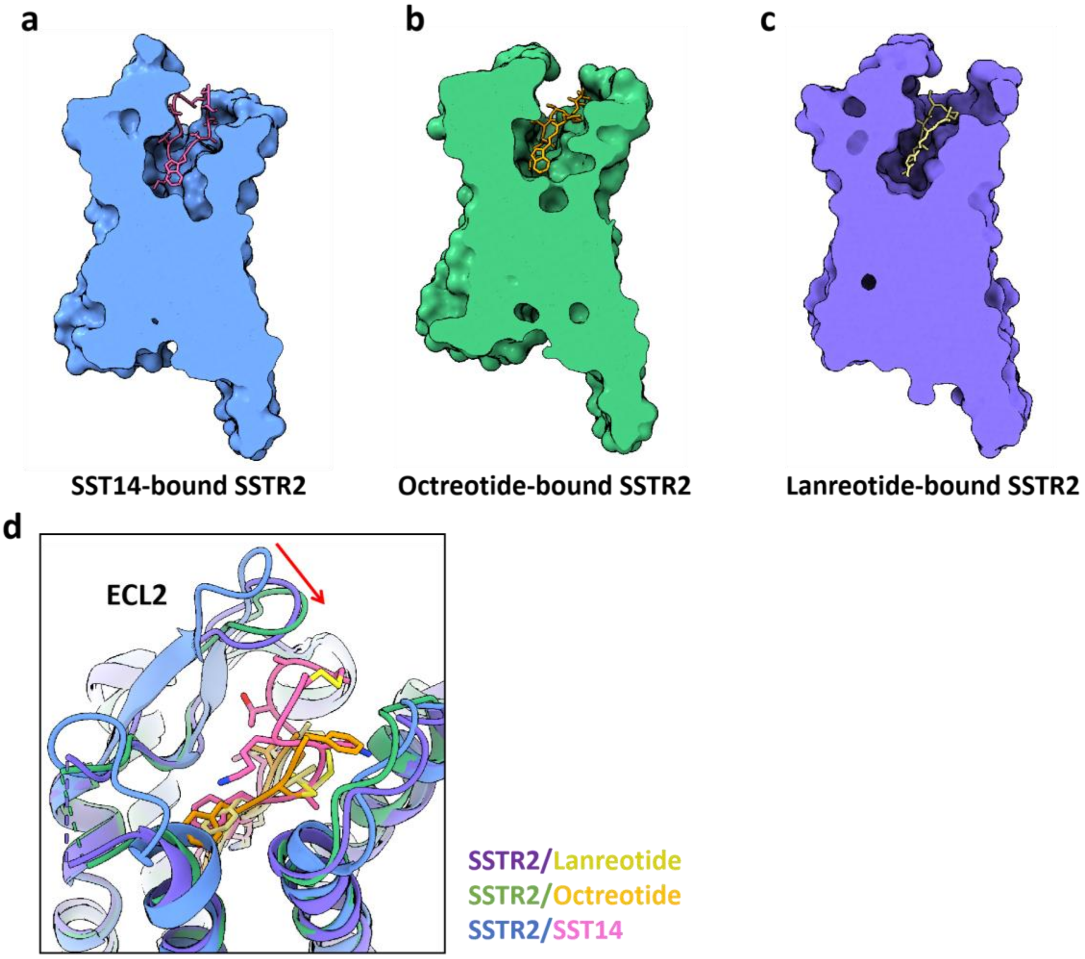

Fig. S6 The comformational change of ECL2 led to the difference between lanreotide, ortreotide and SST14 binding pockets.

a-c Space-filling models and cross-section views of SST14-bound SSTR2**(a)**, ortreotide-bound SSTR2**(b)** and lanreotide-bound SSTR2**(c)**.

d Comparision of ECL2 of lanreotide or ortreotide or SST14 bound SSTR2.

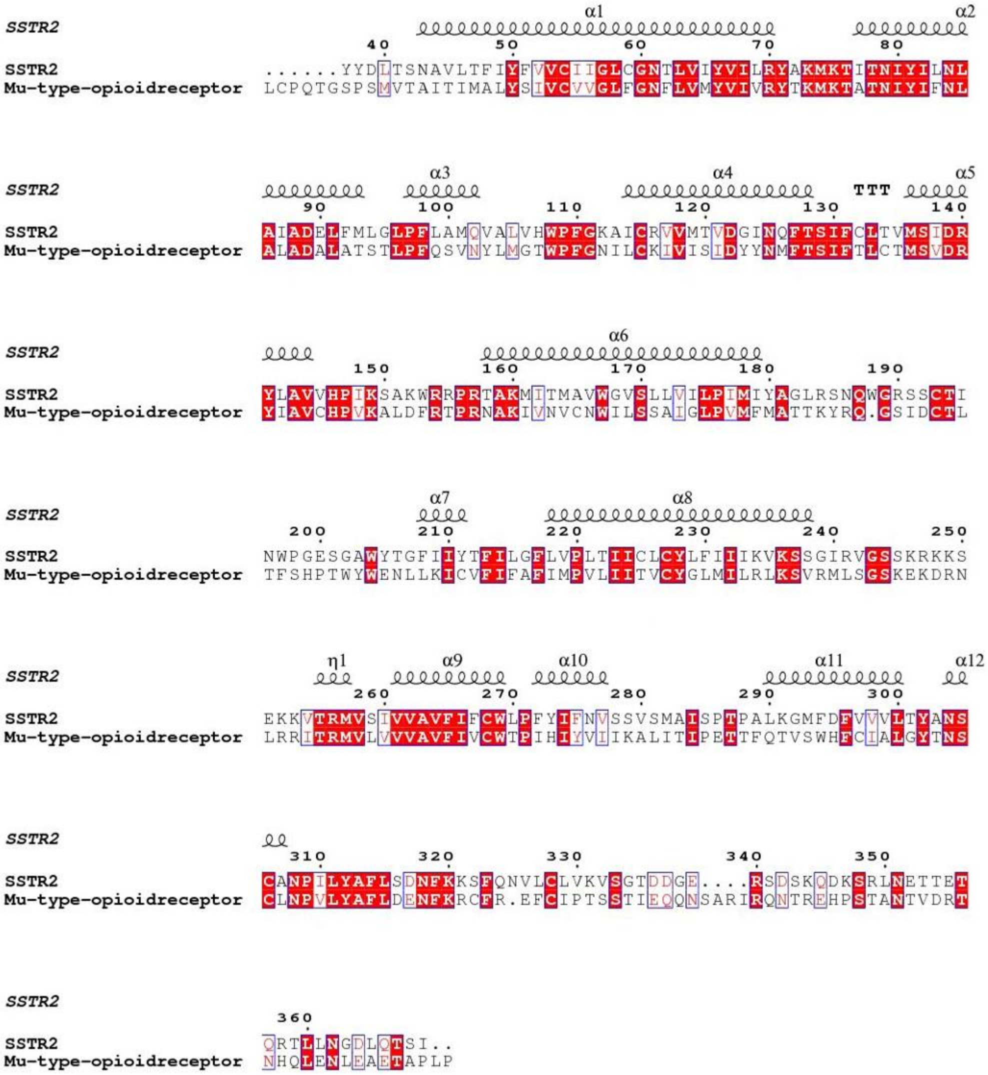

Fig. S7 Sequence alignment of SSTR2 and μ-opioid receptor.

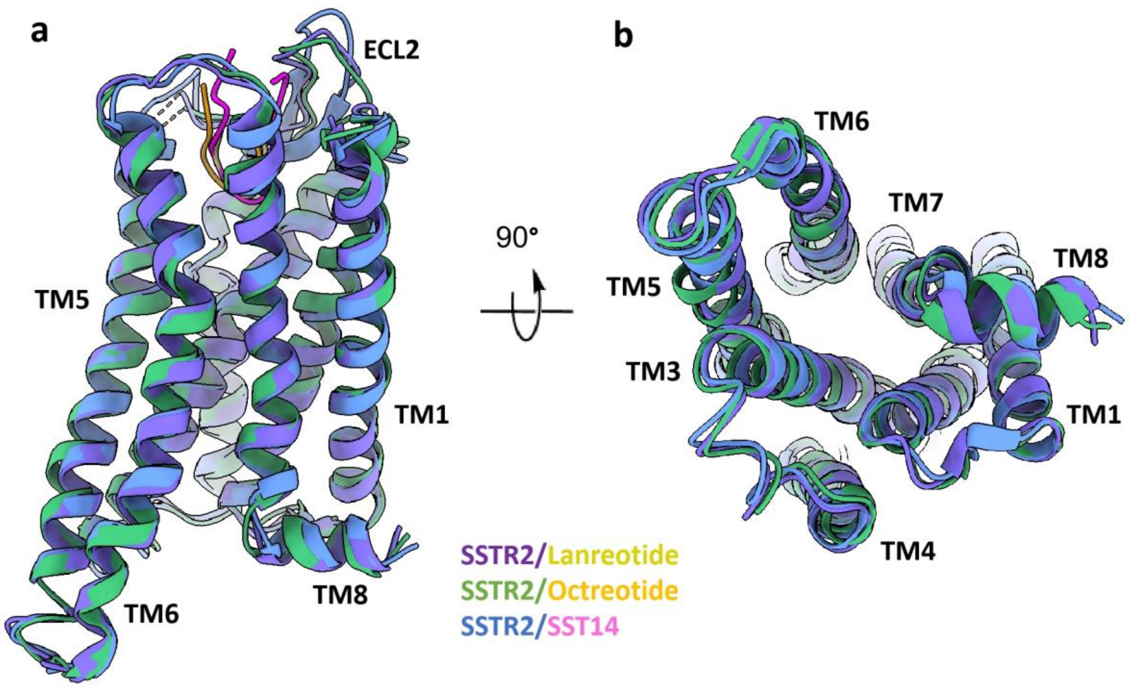

Fig. S8 Conformational comparison of SSTR2 upon activation by different ligands.

Side **(a)** and top **(b)** views of alignment of active SST14-bound SSTR2 (blue), ortreotide-bound SSTR2(green) and lanreotide-bound SSTR2(purple).

Table S1 | Statistics of cryo-EM data collection, 3D reconstruction and model refinement.

**Data Collection**			
**Protein**	SSTR2/SST14	SSTR2/OCT	SSTR2/LAN
**Microscope**	FEI Titan Krios	FEI Titan Krios	FEI Titan Krios
**Voltage (kV)**	300	300	300
**Detector**	Gatan K3 Summit	Gatan K3 Summit	Gatan K3 Summit
**Detector mode**	Super	Super	Super
**Pixel size (Å)**	0.535	0.535	0.535
**Defocus range (μm)**	-1.2 ~ -2.2	-1.2 ~ -2.2	-1.2 ~ -2.2
**Electron dose (e^-/Å^2)**	55	55	55
**Frames per image**	30	30	30
**Exposure time (s)**	3.5	3.5	3.5
**Number of images**	4,189	4,837	7,377
**3D reconstruction**			
**Final Particle number**	457,712	666,575	743,506
**Symmetry**	C1	C1	C1
**Overall resolution (Å)**	2.85	2.97	2.87
**Model refinement**			
**Model composition**			
**Chains**	6	6	6
**Ligands**	DTR:1	DTR:1	DTR:1
**Non-hydrogen atoms**	8,440	8,443	8,410
**Protein residues**	1,152	1,189	1,140
**Bonds (RMSD)**			
**Length (Å)**	0.014	0.011	0.009
**Angles (°)**	1.252	1.189	1.051

**Ramachandran plot (%)**			
**Outliers**	0.09	0.00	0.00
**Allowed**	7.23	8.12	8.30
**Favored**	92.68	91.88	91.70
**Rotamer outliers (%)**	0.83	1.17	0.71
**MolProbity score**	1.81	1.94	1.93
**Clash score**	6.35	7.24	7.84

Table S2 | Summary of EC50 values, Emax for the wild-type SSTR2 and SST22 mutants activation by SST14, Octreotide and Lanreotide. pEC50 values represent the negative logarithm of agonist concentration that produces half maximal response. Emax values are maximal response as percentage of wild-type SSTR2 response. All values are expressed as mean ± s.e.m. of three independent experiments conducted in triplicate. Data were performed using one-way analysis of variance followed by Dunnett's test using wild-type receptors as the control (*P < 0.05, **P < 0.01, ***P < 0.001, ****P < 0.0001). n.d., not determined.

	Ligand	Ca^{2+} pEC_{50}	Ca^{2+} Emax	Surface expression (%WT)
SSTR2 WT	Octreotide	7.7 ± 0.04, (22)	/	100
	SST14	8.0 ± 0.1, (20)	/	
	Lanreotide	7.3 ± 0.05, (10)	/	
SSTR2(1-359)-Largebit	Octreotide	7.5 ± 0.1, (3)	82.6 ± 7.4**	113.5 ± 7.1
	SST14	7.9 ± 0.1, (3)	77.8 ± 6.1****	
	Lanreotide	7.2 ± 0.1, (3)	101.8 ± 9.1	
Q102A	Octreotide	5.9 ± 0.2, (3) ****	103.8 ± 12.1	47.1 ± 1.7
	SST14	7.2 ± 0.2, (3) *	95.3 ± 6.0	
	Lanreotide	6.4 ± 0.1, (3) ****	56.1 ± 4.2****	
D122A	Octreotide	n.d. (3)	5.6 ± 0.5****	114.3 ± 12.2
	SST14	n.d. (3)	6.2 ± 0.3****	
	Lanreotide	n.d. (3)	11.7 ± 4.3****	
Q126A	Octreotide	7.0 ± 0.1, (3) ****	83.5 ± 5.9*	35.9 ± 1.3
	SST14	7.0 ± 0.1, (3) **	92.9 ± 8.1	
	Lanreotide	n.d. (3)	25.2 ± 1.8****	
F272A	Octreotide	n.d. (3)	n.d.	55.5 ± 6.5
	SST14	n.d. (3)	n.d.	
	Lanreotide	n.d. (3)	12.38 ± 1.1****	
F208A	Octreotide	5.9 ± 0.1, (3) ****	98.7 ± 6.9	68.6 ± 8.5
	SST14	6.8 ± 0.1, (3) ***	97.9 ± 3.6	
	Lanreotide	n.d. (3)	27.9 ± 4.0****	
N276A	Octreotide	n.d. (3)	22.3 ± 5.4****	52.7 ± 5.7
	SST14	6.4 ± 0.1, (3) ****	67.7 ± 2.4****	
	Lanreotide	n.d. (3)	13.8 ± 1.6****	
Y302A	Octreotide	n.d. (3)	13.7 ± 3.2****	37.2 ± 3.5
	SST14	n.d. (3)	28.3 ± 4.4****	
	Lanreotide	n.d. (3)	14.0 ± 2.2****	
Q102S	Octreotide	5.9 ± 0.1, (3) ****	91.9 ± 6.5	80.1 ± 2.1
	Lanreotide	6.3 ± 0.1, (3) ****	54.8 ± 1.6****	
F294S	Octreotide	n.d. (3)	n.d.	192.5 ± 5.3
	Lanreotide	n.d. (3)	11.45 ± 0.8****	

N276Q	Octreotide	n.d. (3)	17.9 ± 2.9****	4.0 ± 0.3
	Lanreotide	n.d. (3)	17.0 ± 2.6****	
